# Nanosecond laser-induced surface damage and its mechanism of CaF_2_ optical window at 248 nm KrF excimer laser

**DOI:** 10.1038/s41598-020-62469-y

**Published:** 2020-03-26

**Authors:** Xin Li, Xian-an Dou, Hong Zhu, Yue Hu, Xi Wang

**Affiliations:** 1 State Key Laboratory of Pulsed Power Laser Technology, National University of Defense Technology, Hefei, 230037 China; 2 Anhui Laboratory of Advanced Laser Technology, Hefei, 230037 China; 3 Key Laboratory of Polarization Imaging Detection Technology of Anhui Province, Army Artillery and Air Defense Academy, Hefei, 230037 China; 4 Hefei Second Sanatorium for Retired Cadres of Anhui Province Military Command, Hefei, 230061 China

**Keywords:** Materials for optics, Optical materials and structures, Optical techniques

## Abstract

Calcium fluoride (CaF_2_) crystals is a kind of important optical material for ultraviolet (UV) and deep-ultraviolet (DUV) lithography and high-power laser-related applications. However, its laser-induced damage threshold (LIDT) directly affects the laser power, so that the above-mentioned applications could be limited. Therefore, the research on the damage characteristics and laser damage resistance of CaF_2_ crystals is urgent. A 3D Finite-Difference Time-Domain (FDTD) method with Maxwell spinor equation is used, and the results show that the electric field intensity of rear surface is larger than that of front surface, which causes a lower threshold and is consistent with the experimental observations. And a thermo-mechanical coupled finite element model (FEM) of CaF_2_ with Ce_2_O_3_ impurities, which are introduced by polishing process, has semiquantitatively described the damage mechanism of CaF_2_ by 248 nm-excimer laser.

## Introduction

Due to the large band gap energy (12.1 eV), high transmission at UV range, very low value of nonlinear refractive index, good optical isotropy and excellent chemical stability^1–5^, CaF_2_ optical window materials have been widely used. The results of surface damage of different types of CaF_2_ materials induced by different laser parameters showed that the damage behavior was closely dependent on the materials characteristics as well as laser parameters^6–13^.

Among the works of ultraviolet excimer laser damage of ultraviolet windows, fused silica has been reported mostly^14–24^. In contrast, the reports on fluoride windows are relatively few, and most of the studies were just focused on the damage of fluoride films. Only a few reports are limited to the laser damage of single fluoride windows at 1064 nm wavelength^25^, and most of them were concerned with the damage caused by the fundamental 1064 nm laser and triple frequency 355 nm ultraviolet laser. There are few publications about the damage caused by shorter wavelength excimer laser, such as 248 nm laser. Because ultraviolet excimer lasers present favorable characteristics such as high photon energy, high coupling efficiency as well as high peak power^26–30^, they are expected to be widely used in precision laser machining and military area. This research will provide great significance in deeply understanding the interaction between ultraviolet laser and material, offer important reference in laser precision machining and laser attack-defense field, and also provide theoretical basis for the anti-laser damage ability of optical components.

Based on the self-developed PLD-50 excimer laser, a set of LIDT testing system was established. The CaF_2_ samples were divided into 2 groups, one group was highly polished, and the other one was roughly polished. First, the structural defects from polishing the CaF_2_ crystal were characterized with fluorescence spectroscopy.

Second, the samples were irradiated in a 1-on-1 mode with increasing energy density. Afterwards, the surface fragmentation and damage characteristics of CaF_2_ crystals were analyzed via dark field microscopy. From the experimental results, 2 phenomena were found: (1) the damage threshold of the rear surface of the optical window material is lower than that of the front surface, and (2) the LIDT of highly polished samples is higher than that of roughly polished samples regardless of incident surface or rear surface. The zero-damage thresholds were found from linear fits of the experimental data.

Third, electro-magnetic FDTD and thermo-mechanical FEA simulations were performed to replicate the findings and to get a deeper understanding of the damage process. The lower damage threshold of the rear surface compared to the incident surface is consistent with the FDTD simulation and the approximate value of the damage threshold could be reproduced with a thermo-mechanical FEA simulation. However, the effect that the damage threshold for the rear surface is lower for a roughly polished compared to a highly polished incident surface could not be replicated with the presented simulation schemes.

## Damage tests on CaF_2_ samples

In this paper, when the CaF_2_ samples changes significantly and irreversibly, it is considered to be damaged. In order to reduce the measurement errors, 10 damage points are selected for the same laser fluence, and the damage probability is defined as N_damage/N_total. The zero-probability damage is used as the criterion to measure the LIDT. The damage morphology of the samples was observed and analyzed by optical microscope.

**Figure 1:**
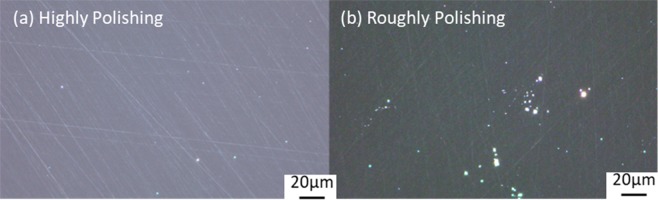
The dark-field images of (**a**) the highly polished samples. (**b**) The roughly polished samples.

In the polishing process of CaF_2_, some physical loads caused by the polishing particles will result in brittle fracture removal, thus brittle defects such as scratches, digs and microcracks will occur on the surface of CaF_2_. These fragmentation defects can induce local modulation of light field during laser irradiation, enhance absorption and also reduce the mechanical strength of component surface. Therefore, these defects are generally considered as a low threshold damage-inducing precursor widely existing on component surface. In this paper, we use dark-field imaging of optical microscopy to characterize the surface fragmentation defects of CaF_2_. In dark-field imaging, the light reflected by the reflecting collector will project on the sample. Due to its great inclination angle, the reflected light cannot enter the objective lens if the sample is a polished mirror, so that only a dark patch can be seen in the eyepiece tube. Figure 1 shows the defects distribution images in dark field of CaF_2_ samples with two different polishing levels. It can be seen that the surface defects of different samples have distinct distribution characteristics under the influence of polishing level. Obvious distribution of defects, mainly scratches and digs, can be seen in roughly polished samples. While in the highly polished samples, scratch is the main defect form, and dig defect is markedly reduced.

**Figure 2:**
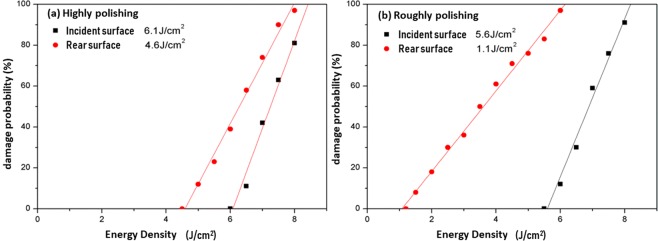
The relationship between damage probability and laser fluence of incident and rear surfaces. (**a**) When the incident surface is under highly polished, the LIDT of incident surface and rear surface are 6.1 J/cm^2^ and 4.6 J/cm^2^, respectively. (**b**) When the polishing level of incident surface is roughly polished, the incident surface LIDT is 5.6 J/cm^2^, and that of rear surface is 1.1 J/cm^2^.

The surface damage curves (linear fitting) of highly polished and roughly polished samples show, reported in Fig. 2, that damage probability increases with the laser fluence. And the LIDT of highly polished CaF_2_ samples, which is 6.1 J/cm^2^, is higher than that of roughly polished samples (5.6 J/cm^2^). By comparing Fig. 2a,b, it can be seen that the LIDT difference between front and rear surfaces of highly polished samples (1.5 J/cm^2^) are much smaller than that of roughly polished samples (4.5 J/cm^2^). What is more, no matter in which sample, the LIDT of rear surface is higher than that of incident surface. And it is worth noting that the polishing levels of the incident surfaces are different, while the rear ones are the same. So the experimental results tells that the polishing level of the incident surface has little influence on the LIDT of the incident surface (6.1 → 5.6 J/cm^2^), but has a great influence on that of the rear surface (5.6 → 1.1 J/cm^2^).

**Figure 3:**
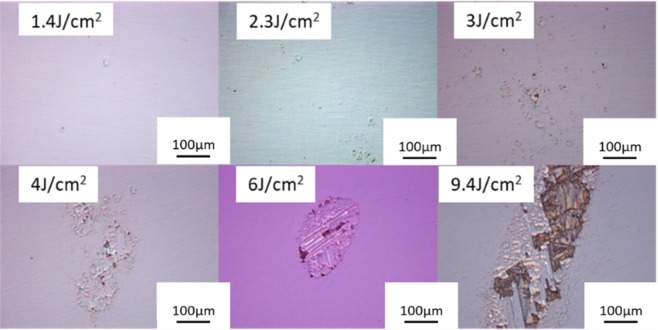
Rear surface damage morphology of roughly polished samples with different laser fluences.

Figure 3 shows the rear surface damage morphology of the samples after roughly polished with different laser fluences. The results show that when the laser fluence is 1.4 J/cm^2^, small damage points appear initially. As the laser fluence increases, the damage area gets bigger. With further increase of the laser fluence a partial or complete peeling of the surface layer occurs.

## Analysis of lower damage threshold on rear surface of CaF_2_ by 3D-FDTD

In the process of laser irradiation, defects on the surface of the sample with the size of laser wavelength level will cause uneven light field distribution in the sample. Many researchers have done a lot of work in theory on the problem of laser induced damage, especially some of them have used finite-difference time-domain (FDTD) method to calculate the light field distribution near the defects. However, the most used is simplified 2D models^31,32^. In practical situation, because of the spatial complexity of defects, 3D-FDTD can better reflect the light field distribution.

**Figure 4:**
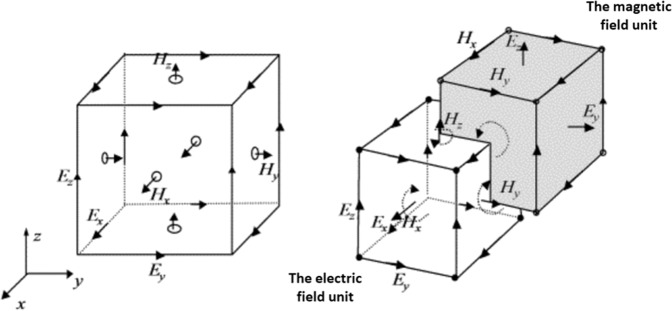
3D Yee’s cell.

The FDTD method is one of the simplest full-wave techniques which can accurately solve a wide range of complicated problems in electromagnetics. However, it generally consumes large computing resources. In other words, it may require a large amount of memory and computation time. The FDTD method uses finite differences as approximations to both the spatial and temporal derivatives that appear in Maxwell’s equations (specifically Ampere’s and Faraday’s laws). The spatial arrangement of each electric field (EF) node and magnetic field (MF) node in FDTD is shown in Fig. 4, which is the famous Yee’s cell^33^. It can be seen from the graph that each MF component is surrounded by four EF components; similarly, each EF component is surrounded by four MF components. According to theoretical calculation and test, this sort of spatial sampling method of electromagnetic field components satisfies both Faraday’s law of electromagnetic induction and Ampere’s circuital law. Moreover, the spatial distribution of electromagnetic field components is also applicable to the differential discretization of Maxwell equation, so as to accurately show the propagation rules of electromagnetic field. In addition, the EF and MF are calculated alternately in time order, and the time intervals are half time steps different from each other. After the Maxwell curl equation is discretized, the explicit difference equation can be formed, so it can be solved iteratively on the time axis without matrix inversion, when the initial value and boundary conditions of a specific electromagnetic field problem are given. The Maxwell curl equations^34^ are

1 $$\nabla \times \overrightarrow{H}=\frac{\partial \overrightarrow{D}}{\partial t}+\overrightarrow{J}$$

2 $$\nabla \times \overrightarrow{E}=\frac{\partial \overrightarrow{B}}{\partial t}+{\overrightarrow{J}}_{m}$$

where ***E*** is the intensity of the EF (V/m), ***D*** is the dielectric flux density (C/m^2^), *H* is the intensity of the MF (A/m), *B* is the magnetic flux density (Wb/m^2^), *J* is the electric current density (A/m^2^), *J*_*m*_ is the magnetic current density (V/m^2^). And the constitutive equations are

3 $$\{\begin{array}{rcl}\overrightarrow{D} & = & \varepsilon \overrightarrow{E}\\ \overrightarrow{B} & = & \mu \overrightarrow{H}\\ \overrightarrow{J} & = & \sigma \overrightarrow{E}\\ {\overrightarrow{J}}_{m} & = & {\sigma }_{m}\overrightarrow{H}\end{array}$$

where *ε* is the dielectric coefficient (F/m), *μ* is the magnetic permeability (H/m), *σ* is the conductivity (S/m), *σ*_*m*_ is the magnetoconductivity (O/m). When calculating scattering problems with FDTD, the computational region is usually divided into the total field area and the scattering field area. Absorption boundary conditions are set outside the scattering field, and a perfectly matched layer (PML) is used. By setting a special dielectric layer at the truncated boundary of the FDTD region, the impedance of the dielectric layer matches that of the adjacent dielectric perfectly, so the incident wave will pass into the PML through the interface without reflection. Because PML is a lossy medium, the transmitted wave entering PML will decay rapidly. Additionally, differ from two-dimensional case, the edges of the total field boundary should also be treated with corresponding boundary treatment.

**Figure 5:**
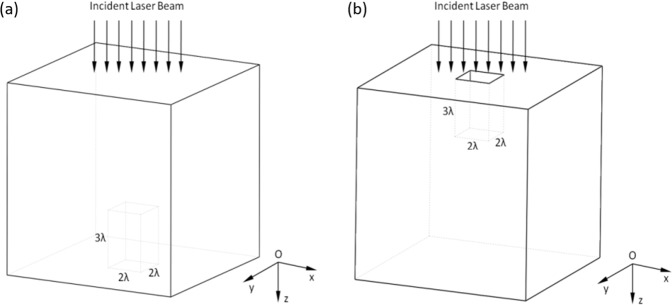
Cubic defect models. (**a**) Defect on the rear surface. (**b**) Defect on the front surface.

The relative dielectric constant of CaF_2_ samples used in this research is 6.81. The incident beam can be regarded as a plane wave with a TM mode (The longitudinal component of the magnetic field is zero, while the longitudinal component of the electric field is not zero) with a wavelength of 248 nm. Suppose that the EF amplitude of the incident wave is 1.0 V/m and its incident direction is along the Z-axis, the front and the rear surfaces of CaF_2_ are parallel to the XOY plane. We consider the defect area to be a vacuum cuboid with a length of 2λ, a width of 2λ, and a height of 3λ. And its position is on the middle of the front surface. The 3D model is shown in Fig. 5. The size of the defect in the two cases is the same. The mesh size is taken as δ = λ/12 = 20.67 nm. In order to save computing resources and make the sample size an integral multiple of δ, we take a part of the CaF_2_ sample as the computational domain. The total field area is set as −6δ~81δ, −6δ~389δ, −16δ~123δ, and the target area is set as 0~72δ, 0~380δ, 0~104δ. The defect area of front surface is 24δ~48δ, 178δ~202δ, 0~36δ, and the defect area of rear surface is 24δ~48δ, 178δ~202δ, 68δ~104δ. The calculation iterations is 1000.

**Figure 6:**
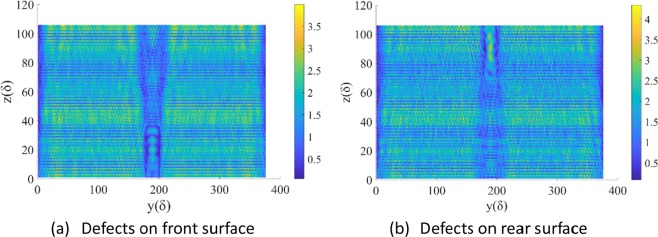
EF intensity distribution on YOZ plane.

**Figure 7:**
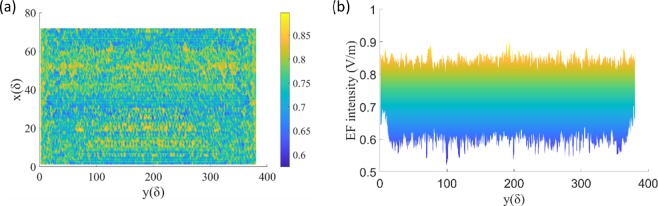
EF intensity distribution of XOY cross-section without defect. (**a**) Z = 0 (front surface). (**b**) EF intensity distribution of front surface along Y-axis when X=36δ.

**Figure 8:**
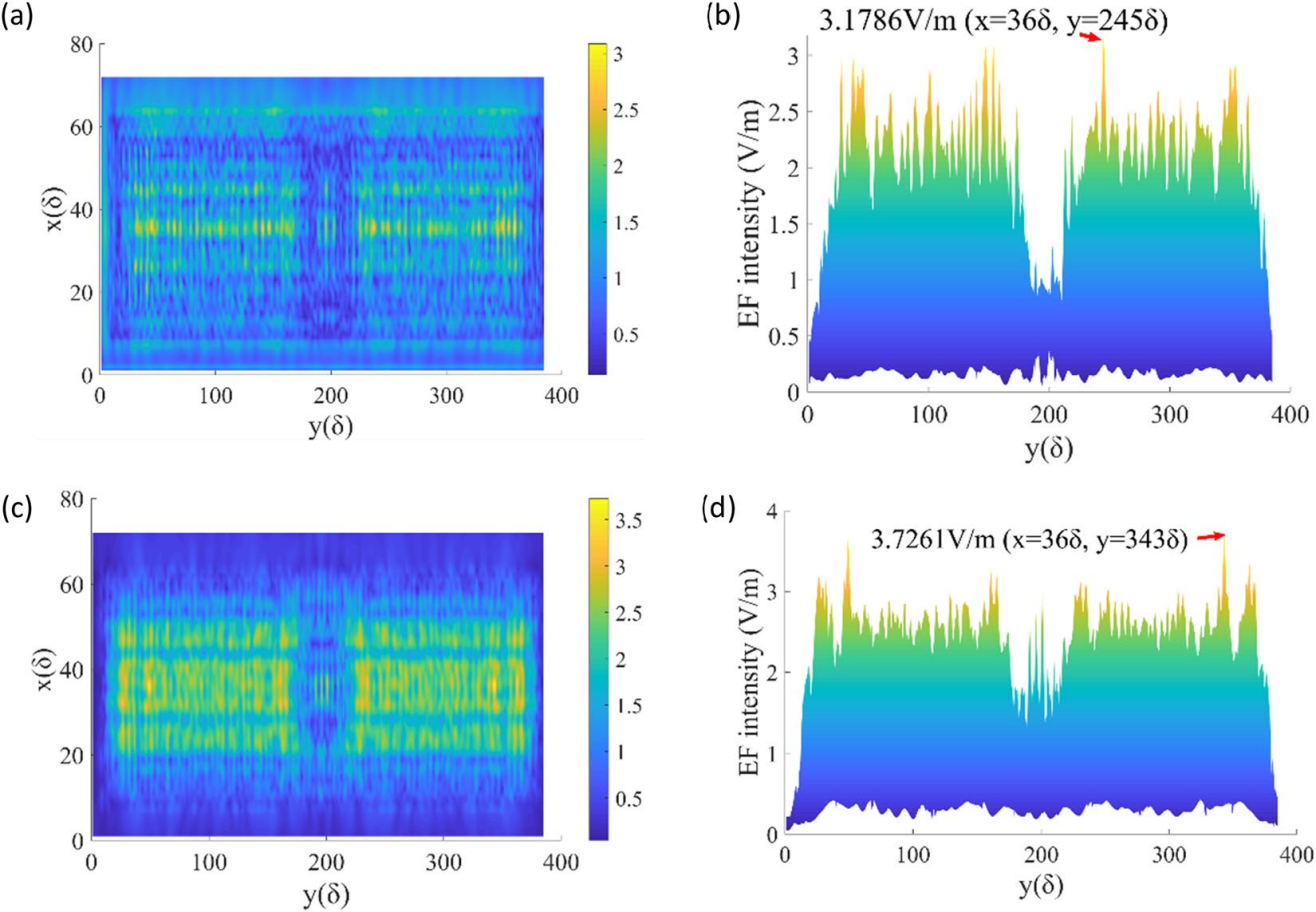
EF intensity distribution of XOY cross-section when the defect is on the front surface. (**a**) Z = 0 (front surface). (**b**) EF intensity distribution of front surface along Y-axis when X = 36δ, and the maximum value is 3.1786 V/m. (**c**) Z = 104δ(rear surface). (**d**) The maximum EF intensity on the rear surface is 3.7261 V/m.

**Figure 9:**
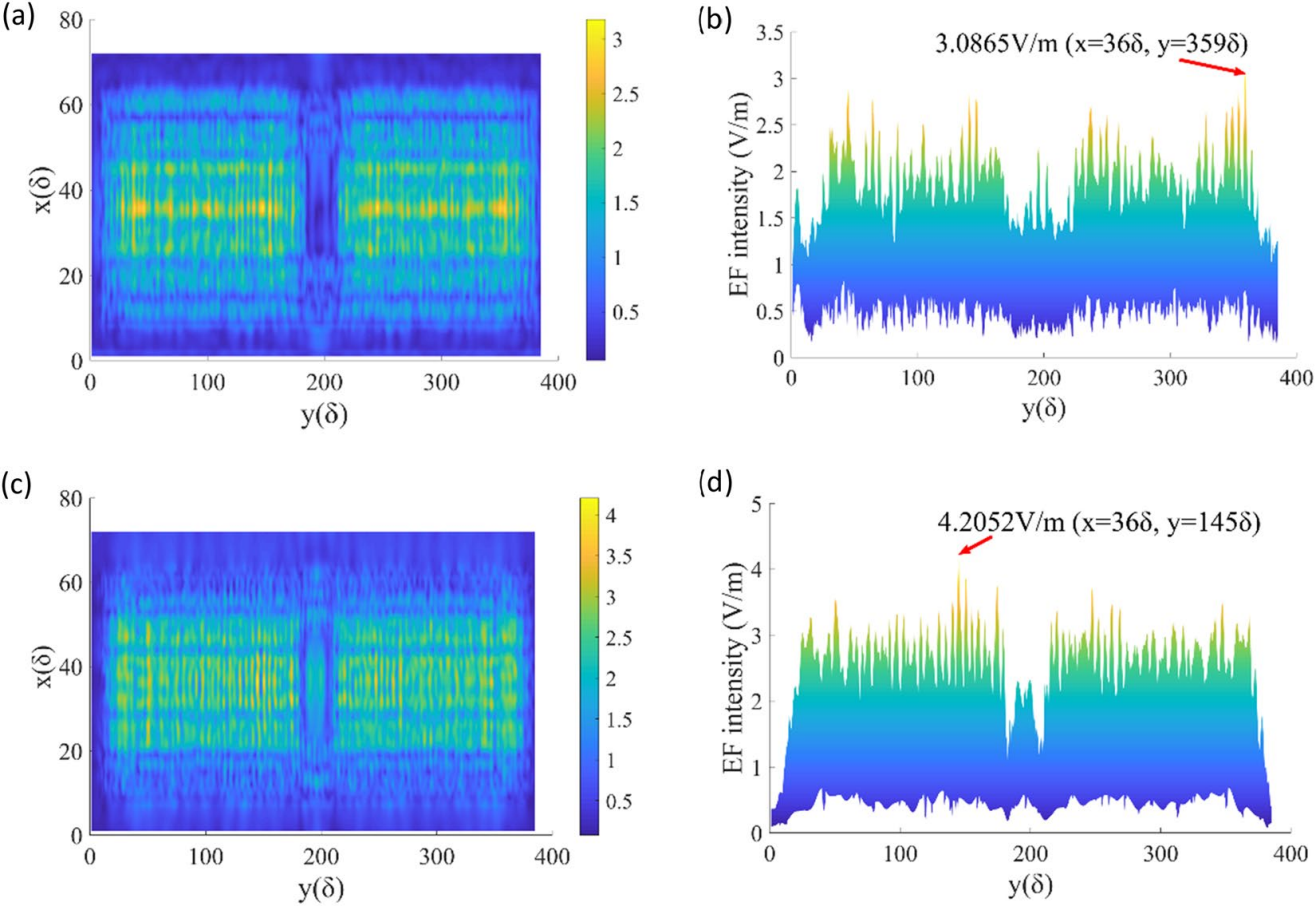
EF intensity distribution of XOY cross-section when the defect is on the rear surface. (**a**) Z = 0 (front surface). (**b**) The maximum EF intensity on the front surface is 3.0865 V/m. (**c**) Z = 104δ (rear surface). (**d**) The maximum EF intensity on the rear surface is 4.2052 V/m.

The relevant 3D-FDTD results are shown in Figs. 6–9. In Fig. 6, the plane of x = 28δ, which is central cross-section of the defect, is selected because the light field on this plane is most obviously modulated by the defect. Since the incident wave is TM mode, all the calculation results of the EF are components in the X direction, i.e., E_x_. Figure 6(a) is the case of defect on the front surface of CaF_2_ (Z = 0 is the incident/front surface). We can see that the EF intensity near the defect is obviously modulated, and the EF distribution near the rear surface changes to some extent. Except for the area from the defect to the rear surface (148δ < Y < 230δ, 36 < Z < 104δ), the EF intensity does not change significantly. The case of defect on the rear surface is shown in Fig. 6(b). The EF distribution near the defect is obviously affected, and the EF intensity near the rear surface is also greatly increased. The above phenomenon can be explained as follows:

For the case of the defect on the front surface. Because of the defect (especially for the size of 2λ × 2λ × 3λ), the scattering of the transmitted laser wave occurs. An obvious standing wave is formed in front of the defect. As a result, behind the defect, the mode of laser is no longer the basic mode of a TE mode, but the superposition and coupling of multiple modes. When the defect is on the rear surface, the situation is similar because the defect has a depth of 3λ.

Also, the distribution of the EF in the XOY plane is investigated. As a reference, EF intensity distribution of XOY cross-section without defect is shown in Fig. 7. The electric field along Y-axis when X = 36δ is evenly distributed between 0.6 V/m and 0.9 V/m. Then, by comparing Figs. 8 and 9, the EF intensity varies with the location of the defect. It can be seen that the EF intensity on the rear surface (Figs. 8c,d and 9c,d) is more or less greater than that near the front surface (Figs. 8a,b and 9a,b) in the range of about 24δ < X < 48δ, which is consistent with the results of Fig. 2.

**Figure 10:**
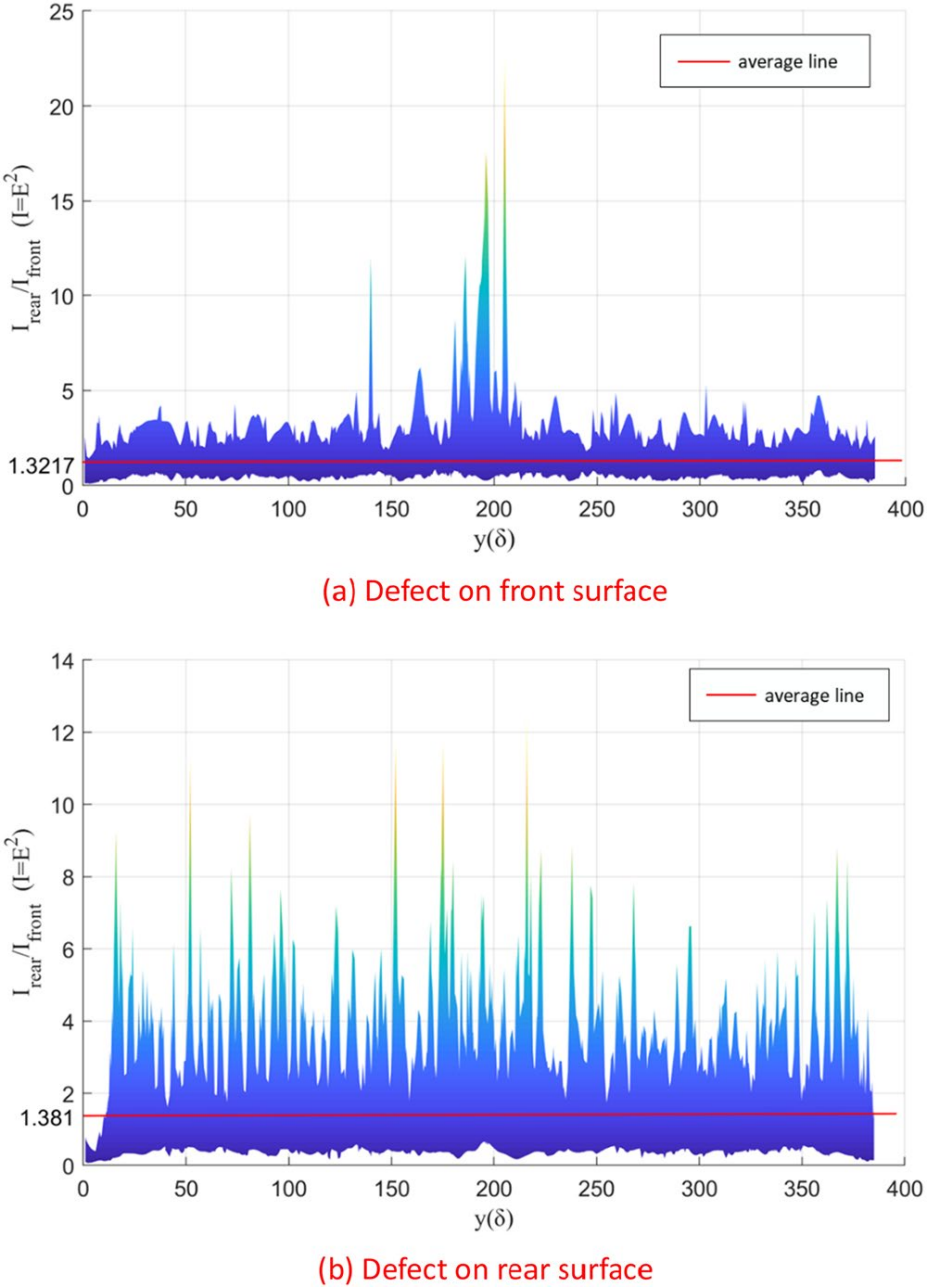
The ratio of the laser intensity (square of electric field strength) of the rear surface to that of the front surface.

However, these are just two special XOY planes. To better illustrate the problem, the difference of EF intensity between front and rear surfaces in two cases are drawn in Fig. 10. The positive value means the EF intensity of rear surface is larger than that of front surface with the same x, y coordinate. It is obvious that the sum of EF intensity difference between front and rear surfaces is far more than zero (5884.3 V/m for the case of defect on the front surface and 7663.1 V/m for the case of defect on the rear surface). And the numbers of pairs of nodes (the corresponding points on front and rear surface, which have the same X, Y coordinate values, but different Z coordinate values) with their EF intensity difference above zero are 6271 (66.01%) and 5514 (58.04%), respectively.

The essence of light is electromagnetic wave, so analyzing the distribution of electromagnetic field in target materials, in certain degree, can reflect the coupling intensity of laser power in materials. However, the distribution of electromagnetic field in target materials is not enough to explain the damage mechanism, namely, the value of damage threshold, it can only analyze the problems of transmission and distribution of electromagnetic fields. As a result, in this paper, the FDTD was used to analyze the relative value of electric fields of the front and rear surface. The comparison result can show which surface couples more energy of the laser, in other words, the surface which couples more energy of laser will have a low damage threshold.

The explanation of the FDTD simulation results is interference. Here the incident wave is added to the reflected waves of the incident surface and the rear surface of CaF_2_ which would cause higher field strength and higher energy density via constructive interference. However, because this happens in air (which cannot get damaged) at the incident surface and inside the material at the rear surface, the rear surface gets damaged at lower energies.

We assume that the electric field strength on the rear *E*_*r*_ is enhanced by a factor *A* due to reflection when compared to the incident E-field *E*_*i*_.

4 $${E}_{r}=A\times {E}_{i}$$

The electric field on the front *E*_*f*_ is assumed to be equal to the incident field *E*_*i*_

5 $${E}_{f}={E}_{i}$$

We also assume that there is a damage threshold E-Field *E*_*D*_, which represents the field strength just before the material gets damaged. We now irradiate with an incident E-Field *E*_*i1*_, which causes a damage threshold E-field to appear on the rear:

6 $${E}_{r}={E}_{D}=A\times {E}_{i1}$$

Now we irradiate with an incident E-field *E*_*i2*_ which causes damage threshold E-field to appear on the front.

7 $${E}_{f}={E}_{D}={E}_{i2}$$

Now we divide Eq. (6) by Eq. (7),

8 $$\frac{{E}_{r}}{{E}_{f}}=A\times \frac{{E}_{i1}}{{E}_{i2}}=1$$

where *E*_*f*_ = *E*_*r*_ = *E*_*D*_, and

9 $$A=\frac{{E}_{i2}}{{E}_{i1}}$$

Now we square it and

10 $${A}^{2}={\left(\frac{{E}_{i2}}{{E}_{i1}}\right)}^{2}=\frac{{I}_{i2}}{{I}_{i1}}=1.326$$

where *I* is the laser intensity, which is square of E-field. From Fig. 2 we know *I*_*i2*_ corresponds to the LIDT of front surface, and *I*_*i1*_ corresponds to the LIDT of rear surface. Here, we take the values in the highly polished case, that is, *I*_*i2*_ is 6.1 J/cm^2^ and *I*_*i1*_ is 4.6 J/cm^2^.

Simulation results in Fig. 10 could successfully replicate the finding. $\frac{{{\rm{E}}}_{r}^{2}}{{E}_{i}^{2}}$ values are obtained by calculating the ratio of electric field strength of the corresponding nodes (the corresponding points on front and rear surface, which have the same X, Y coordinate values, but different Z coordinate values), and then square them. Figure 10a,b are the cases that the defect is on the front surface and the rear surface respectively. It can be seen that average and standard deviation values of *A*^2^ are 1.3217, 1.381 and 0.3826, 0.3346 in 2 cases, and 1.326 lies in that range.

With the coupling of the laser power, electromagnetic energy is converted into heat and mechanical energy, which are the direct reasons of damaging. So in the next FEA part, the laser is regarded as a kind of heat source. Through analyzing the heat and force effect of the physical process, the value of damage threshold can be predicted.

## Thermo-mechanical coupling FEA model of laser induced damage to CaF_2_

**Figure 11:**
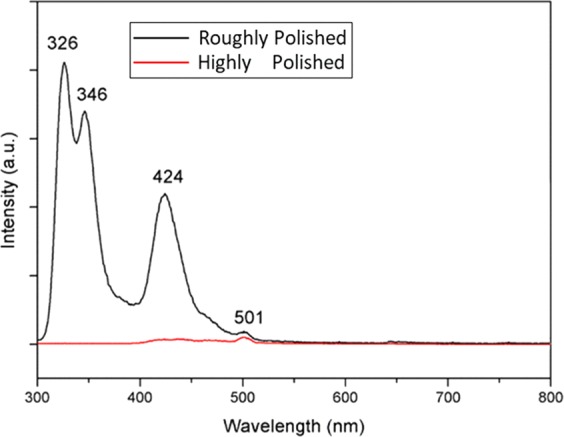
Fluorescence spectrum of CaF_2_ substrates.

Figure 11 shows the photoluminescence spectrum of CaF_2_ samples. A 250 nm-wavelength Xe lamp is used as excitation source, and the detection spectrum ranges from 300 to 800 nm. It can be seen that the luminescence signal in highly polished sample is rather weak, with only a weak peak at 501 nm, while in roughly polished sample there are four emission peaks at 326 nm, 346 nm, 424 nm, and 501 nm. From the peak wavelength and spectral bandwidth of emission peaks, it can be confirmed that 326 and 346 nm emission are originated from 4f → 5d transition emission of Ce^3+^, and 424 nm emission is originated from 4f → 5d transition emission of Eu^2+^. However, 501 nm emission was unclear. This indicates that roughly polished samples contain trace impurity ions. Comparatively, the impurity content in highly polished samples is very low. It should be noted that The purpose of polishing is to minimize the content of impurities on the surface, but no residue at all is impossible. From Fig. 1a, some bright spots (impurity particles or dig defect) still can be seen.

**Figure 12:**
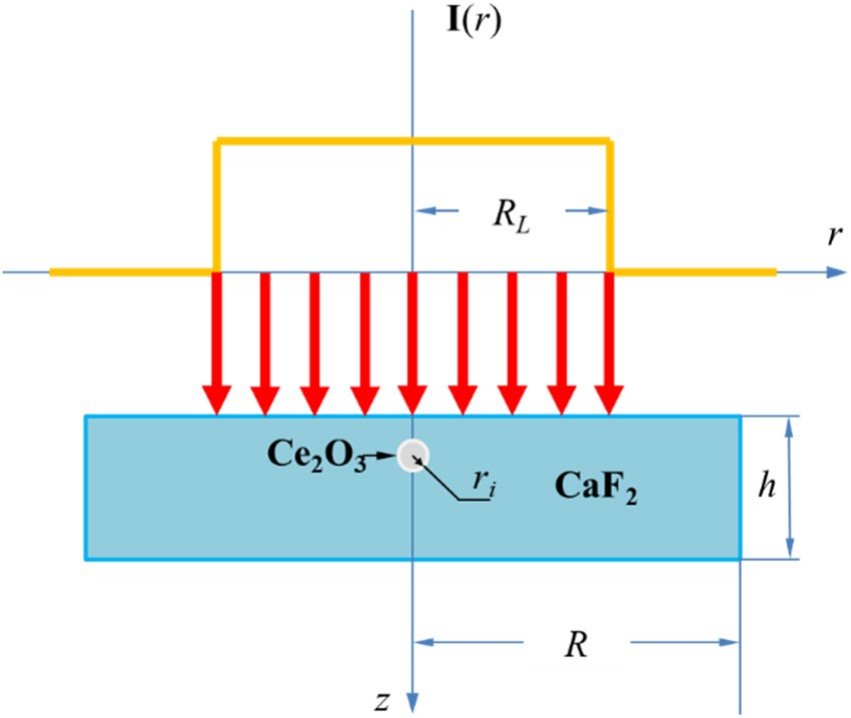
Principle model of CaF_2_ irradiated by an ultraviolet excimer laser.

**Figure 13:**
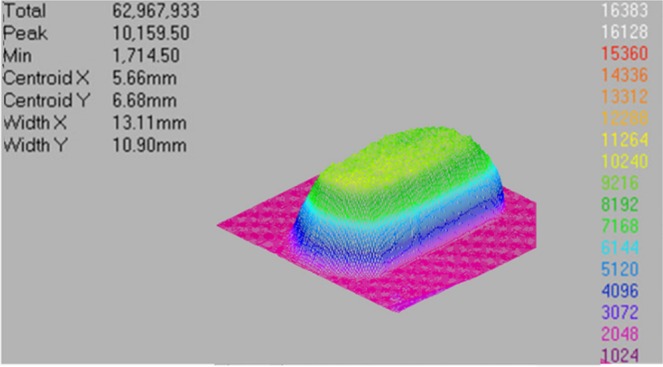
Energy distribution (2D and 3D) of the KrF laser spot.

In order to calculate the temperature and stress fields of CaF_2_ irradiated by 248 nm laser, the theoretical model is established and shown in Fig. 12 (2D crosssection of the model). The laser focus is on the CaF_2_ surface, and the duration of the pulse is *τ* = 20 ns. The CaF_2_ sample is disc-shaped, and the laser is vertically incident to the surface of the sample (*Z* = 0), the center of the laser beam is concentric with the sample center. In order to save computing resources, we take a part of the CaF_2_ sample as the computational domain. The radius and thickness of the CaF_2_ model are *R* = 2 mm and *h* = 1 mm, respectively. And the Ce_2_O_3_ impurity particle is a *r*_*i*_ = 300 nm sphere, its spherical center is 1μm away from the incident surface of the laser. Figure 13 shows the energy distribution of the KrF laser spot by a beam quality analyzer. It shows that the output laser is a flat-topped beam, and the energy distribution is similar to uniform distribution, which is different from the Gaussian distribution of general lasers. Therefore, in this paper, the temporal distribution of the laser beam is assumed as a step function and the peak power density *I*_0_ is uniform in pulse duration.

The absorbed energy in CaF_2_ can be expressed by the heat conduction equation^30^,

11 $$\rho {C}_{p}\frac{\partial }{\partial t}T(r,z,t)=\nabla \cdot [k\nabla T(r,z,t)]+Q(r,z,t)$$

12 $$-k{\frac{\partial T}{\partial n}|}_{z=h}=-\,k{\frac{\partial T}{\partial n}|}_{r=R}=0$$

13 $$T(r,z,0)={T}_{0}$$

where Eqs. (12) and (13) are the boundary and initial conditions, respectively. *C*_*p*_, *ρ*, and *k* are isobarically heat capacity, density, and thermal conductivity, respectively. *T*_0_ = 293 K is an initial temperature, *Q(r, z, t)* is the heat source, which can be shown as:

14 $$Q(r,z,t)=I(r,t)\cdot (1-{R}_{s})\cdot \alpha \cdot \exp (-\alpha z)$$

where *α* is the absorption coefficient of the material and *R*_*s*_ is the surface reflectance of CaF_2_. *I*(*r, t*) is the incident laser intensity, which can be written as:

15 $$I(r,t)={I}_{0}\cdot f(r)\cdot g(t)$$

where *I*_0_ is the peak power density of the incident laser. *f*(*r*) and *g*(*t*) are the spatial and temporal distribution functions of the laser, respectively, and they can be described as follows:

16 $$f(r)=\{\begin{array}{ll}1,\, & 0\le r\le {R}_{L}\\ 0, & r > {R}_{L}\end{array}$$

17 $$g(t)=\{\begin{array}{ll}1, & 0 < t < \tau \\ 0, & t > \tau \end{array}$$

where *R*_*L*_ is the beam radius and *τ* is the pulse duration. In this paper, the effect of thermal radiation and thermal convection can be ignored since the duration of the laser pulse is very short.

Related parameters of CaF_2_ and Ce_2_O_3_ are listed in Tables 1 and 2. The transmission of the umcoated CaF_2_ sample at 248 nm is R_s_ = 92.28%^35^. In the analysis model, because CaF_2_ is the window of 248 nm, we consider that 92.28% laser influece is absorbed by the Ce_2_O_3_ particle.

**Table 1:** Parameters of CaF_2_ for analysis^36^.

Property	Value
Density (kg/m^3^)	3180
Specific heat (J/kg·K)	911
Thermal conductivity [W/m·K]	9.71
Young modulus (GPa)	110
Poisson ratio	0.29
Melting point (K)	1635
Linear expansibility (K^−1^)	18.85 × 10^−6^
Compressive strength (MPa)	300
Tensile strength (MPa)	34

**Table 2:** Parameters of Ce_2_O_3_ for analysis.

Property	Value
Density (kg/m^3^)	7300
Specific heat (J/kg·K)	380
Thermal conductivity [W/m·K]	23
Young modulus (GPa)	200
Poisson ratio	0.3
Linear expansibility (K^−1^)	16 × 10^−6^

**Figure 14:**
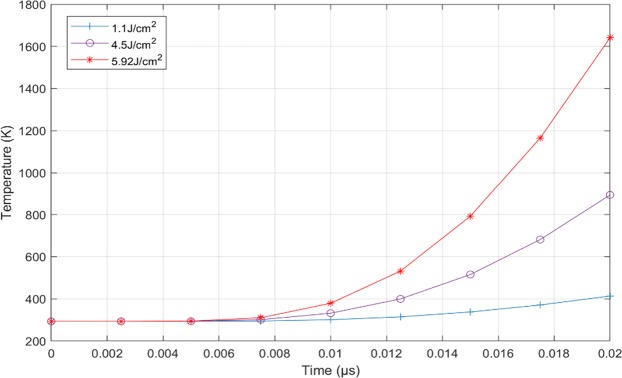
The maximum temperature of incident surface at 1.1 J/cm^2^, 4.5 J/cm^2^, and 5.92 J/cm^2^.

**Figure 15:**
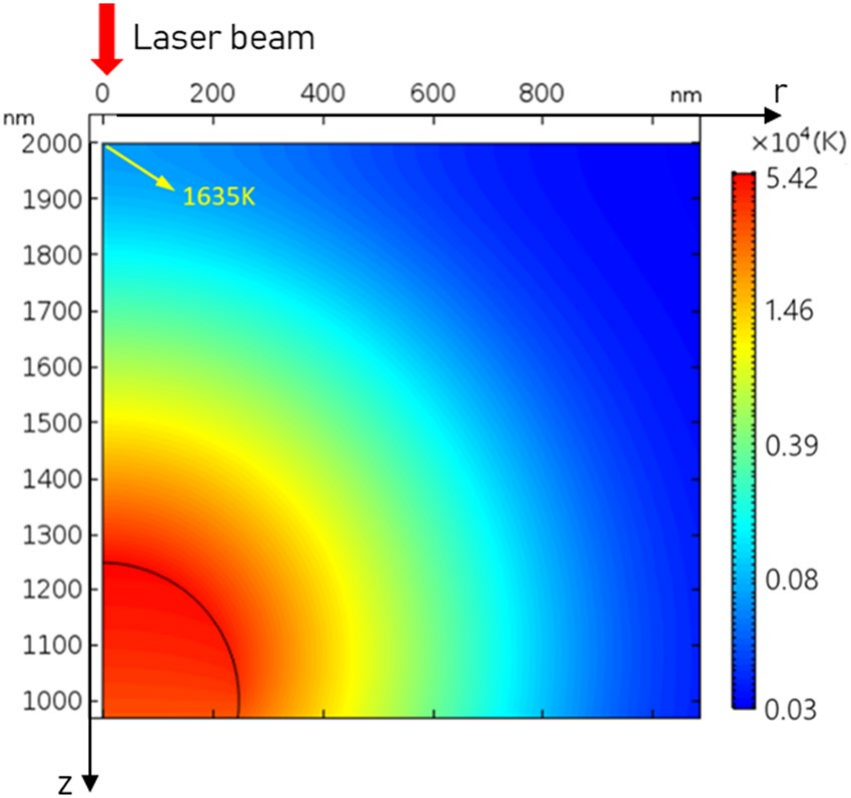
The temperature distribution of CaF_2_ with Ce_2_O_3_ particle at 5.92 J/cm^2^.

**Figure 16:**
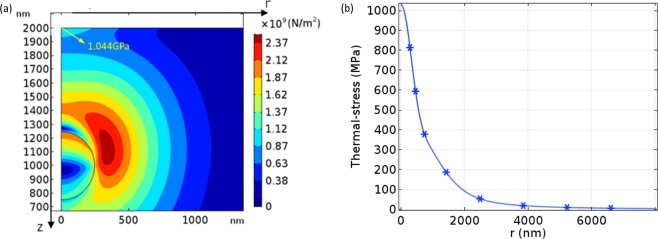
The thermal-stress distribution of CaF_2_ with Ce_2_O_3_ particle at 5.92 J/cm^2^. (**a**) In the sample. (**b**) On the incident surface.

In this paper, due to the extremely short time scale, we consider that the melting damage occurs when the incident surface of the CaF_2_ sample reaches its melting point. Figure 14 shows the maximum temperature on incident surface of the CaF_2_ sample with different laser fluences. It can be seen that the maximum temperature of incident surface just reaches the melting point of 1635K when the laser fluence is 5.92 J/cm^2^ (Fig. 15), and the melting damage will occur in the laser spot area. So 5.92 J/cm^2^ is the melting damage threshold of CaF_2_. However, the corresponding maximum thermal-stress at 5.92 J/cm^2^ is 1.044 GPa (Fig. 16), which is far more than the compressive strength of CaF_2_ (300 MPa). So, it is obvious that the compressive stress damage occurs before the melting damage.

**Figure 17:**
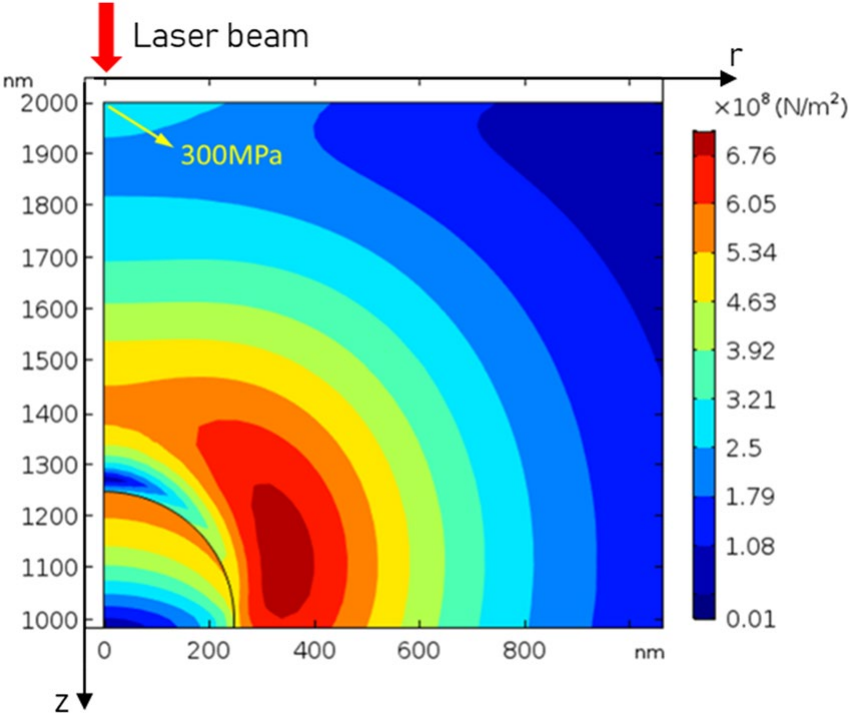
The thermal-stress distribution of CaF_2_ with Ce_2_O_3_ particle at 3.74 J/cm^2^.

**Figure 18:**
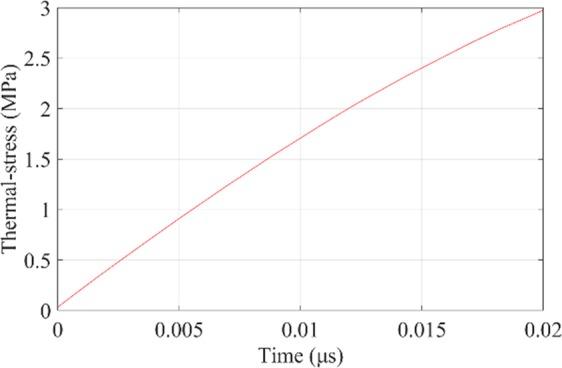
The maximum thermal-stress of incident surface at 3.74 J/cm^2^.

**Figure 19:**
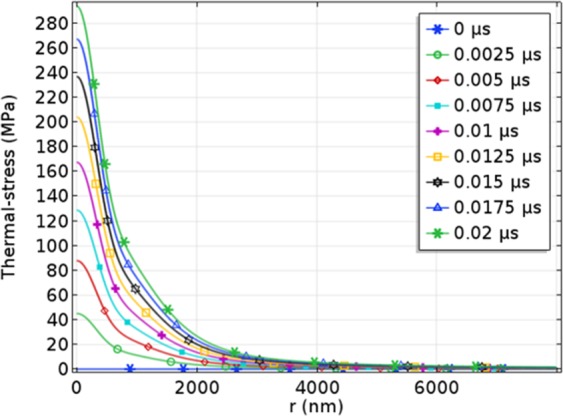
Thermal-stress distribution of incident surface at 3.74 J/cm^2^.

Figure 17 shows the thermal-stress distribution between the incident surface and Ce_2_O_3_ particle with laser fluence of 3.74 J/cm^2^. And Figs. 18 and 19 show the maximum stress value and the stress distribution along r-axis at different time, respectively. It can be seen that the compressive stress at the spot center just reaches the compressive strength (300 MPa) at 20 ns, so the compressive stress damage on the surface exactly occurs. However, compared with the experimental results, the calculated stress damage threshold (3.74 J/cm^2^) is between the measurements of rear surface with roughly polished (1.1 J/cm^2^) and highly polished (4.6 J/cm^2^). Through analysis, the reason is (1) in roughly polished condition, the distribution density of Ce_2_O_3_ particles is larger than the calculation model, while in highly polished condition, the distribution density of Ce_2_O_3_ particles is smaller than the calculation model; (2) there are some other impurities like EuO_x_ in the CaF_2_ samples, and the parameters are not reported.

## Conclusions

To summarize, laser-driven surface damage and material failure behavior of CaF_2_ crystal under ultraviolet laser beam irradiation have been systematically studied. Experimental research on damage in CaF_2_ irradiated by a 248 nm/20 ns KrF excimer laser is carried out. The 3D-FDTD simulation could successfully replicate the finding that the rear surface is always damaged before incident surface by showing that the laser intensity (square of electric field strength) on the rear is always approximately 1.3–1.4 times higher than that on the front (from Fig. 10, the ratios are 1.3217 and 1.381, respectively). The phenomenon that the rear surface always gets damaged at lower energies, when the front surface is roughly polished compared to highly polished, could not be reproduced. This might be achievable with an increase in the computational grid to the width of the sample window. Because in the present simulation we are still in the near field.

In addition, this paper essentially establishes the theoretical model of nanosecond laser damage to optical window materials by FEA method. Due to the unknown distribution density of Ce_2_O_3_ in CaF_2_, the analytical results are somewhat different from the experimental results. However, the presented model could successfully reproduce the order of magnitude of the laser induced damage threshold.

## Methods

### Laser system and samples

**Figure 20:**
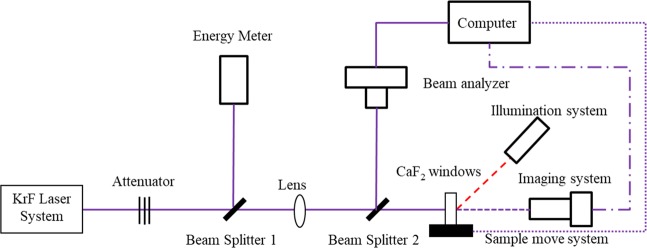
Experimental setup of irradiation system (a 248 nm KrF laser facility was used).

The experimental optical path of CaF_2_ windows damaged by 248 nm KrF ultraviolet laser is shown in Fig. 20. The laser source is a 248 nm KrF excimer laser, which was produced by Anhui Institute of Optics and Fine Mechanics, Chinese Academy of Sciences. It can emit high-quality pulsed laser beams with adjustable frequency (1 Hz to 50 Hz), and pulse width is 20 ns, and its single pulse energy can be adjusted in the range of 0mJ to 550mJ. An OPHIR L30A-10MM energy meter is used to monitor the pulse energy. The CaF_2_ window samples with both surfaces polished is 30 × 30 × 3 mm in size. “1-on-1” mode is adopted in the experiment, i.e., each position on the sample is allowed no more than one pulse shot on it.

### Materials

Commercially available CaF_2_ (Beijing Scitlion Technology Corp., LTD.) was used in this study. And the polishing of samples is also done by Beijing Scitlion Technology Corp., LTD.

### Numerical simulations

The EF distribution in CaF_2_ samples when the defect is on the incident surface and rear surface is calculated by FDTD Solutions. And the temperature and thermal-stress distribution in CaF_2_ samples with Ce_2_O_3_ particle after irradiation by 248 nm KrF laser are calculated using COMSOL Multiphysics.
